# The Effect of Maternal Death on the Health of the Husband and Children in a Rural Area of China: A Prospective Cohort Study

**DOI:** 10.1371/journal.pone.0157122

**Published:** 2016-06-09

**Authors:** Hong Zhou, Long Zhang, Fang Ye, Hai-jun Wang, Dale Huntington, Yanjie Huang, Anqi Wang, Shuiqing Liu, Yan Wang

**Affiliations:** 1 Division of Maternal and Child Health, School of Public Health, Peking University, No.38 Xueyuan Road Haidian District, Beijing, China; 2 Department of Epidemiology, Johns Hopkins Bloomberg School of Public Health, Baltimore, Maryland, United States of America; 3 Department of Preventive Health Care, China-Japan Friendship Hospital, Beijing, China No.38 Xueyuan Road Haidian District, Beijing, China; 4 Institute of Child and Adolescent Health, School of Public Health, Peking University, No.38 Xueyuan Road Haidian District, Beijing, China; 5 Asia Pacific Observatory on Health Systems and Policies, World Health Organization Western Pacific Regional Office, Manila, Philippines; 6 Department of Health Policy and Management, Johns Hopkins Bloomberg School of Public Health, Baltimore, Maryland, United States of America; Hunter College, UNITED STATES

## Abstract

**Objective:**

To examine the effects of maternal death on the health of the index child, the health and educational attainment of the older children, and the mental health and quality of life of the surviving husband.

**Methods:**

A cohort study including 183 households that experienced a maternal death matched to 346 households that experienced childbirth but not a maternal death was conducted prospectively between June 2009 and October 2011 in rural China. Data on household sociodemographic characteristics, physical and mental health were collected using a quantitative questionnaire and medical examination at baseline and follow-up surveys. Multivariate linear regression, logistic regression models and difference-in-difference (DID) were used to compare differences of outcomes between two groups.

**Findings:**

The index children who experienced the loss of a mother had a significantly higher likelihood of dying, abandonment and malnutrition compared to children whose mothers survived at the follow-up survey. The risk of not attending school on time and dropping out of school among older children in the affected group was higher than those in the control group during the follow-up. Husbands whose wife died had significantly lower EQ-5D index and EQ-VAS both at baseline and at follow-up surveys compared to those without experiencing a wife’s death, suggesting an immediate and sustained poorer mental health quality of life among the surviving husbands. Also the prevalence of posttraumatic stress disorder (PTSD) was 72.6% at baseline and 56.2% at follow-up among husbands whose wife died.

**Conclusions:**

Maternal death has multifaceted and spillover effects on the physical and mental health of family members that are sustained over time. Programmes that reduce maternal mortality will mitigate repercussions on surviving family members are critical and needed.

## Background

China has made tremendous progress in decreasing maternal mortality rates (MMRs) in the past 25 years [1, 2]. MMR decreased by 72.8% from 80.0 per 100,000 live births in 1991 to 21.7 per 100,000 live births in 2014 [2]. These dramatic reductions were attributed to rapid economic growth and substantial investment in health-care services in China. Despite this progress, regional heterogeneity of MMR existed and maternal deaths still occurred, principally in rural more isolated communities. There were 5,900 maternal deaths in 2013 in China, accounting for 2% of global maternal deaths for the year [3].

There is a consensus that the loss of a mother is not just a grievous single event to the family, but that has large and negative effects on the surviving children, spouse, families and communities [4]. A growing body of evidence has demonstrated the spillover effects of maternal death on children who are left behind. For instance, an Ethiopia longitudinal study found that babies who experienced the loss of a mother within their first 30 days had 46 times higher risk of dying compared to those whose mothers survived [5]. A similar result was found in a study from rural Tanzania, indicating that children orphaned by an early maternal death had a 48% probability of dying before their first birthday compared to 6% for those whose mothers survived [6]. Moreover, the far-reaching impacts of maternal death can extend to other family members. Several qualitative studies in Africa suggested that older children who experienced the loss of a mother were less likely to have educational supervision and psychological support from caregivers, while also assuming additional caregiving responsibilities for the index baby, which made them more likely to drop out of school [7–9]. Economic drains derived from the maternal death (e.g., medical and funeral expenses) can also exacerbate long-term adverse effects on the families [10, 11].

Previous studies undertaken in Africa (described above), have begun to investigate the influence of maternal death on outcomes of children, especially the well-established connection between a maternal death and survival of the index children at their early stage of life. However, generalizability of study results may be limited due to localized cultural effects and being compounded by the high HIV prevalence in some settings. Very little is known on the lasting effects on the index children and their families, particularly the surviving husband. Also, qualitative studies on educational attainment among the older children who experienced the loss of a mother may not sufficient to detect the extent to which their school performances are effected by the loss of their mother.

This study aimed to explore wide-ranging impacts of maternal death on index children, older children and husbands in the context of relatively low MMRs in China.

## Methods

The study was approved by the ethical reviews boards at Peking University and the World Health Organization. Signed informed consent for all caregivers/guardians on behalf of themselves and minor children were obtained prior to any interviews. All the interviews were conducted without any incentives, and timed to take place after an appropriate grieving period had passed.

### Study design

Details regarding the study design have been described elsewhere [11, 12]. Briefly, a prospective cohort study was conducted in the rural regions of Hebei, Henan and Yunnan provinces in China. The study areas were chosen to represent areas with low, moderate and high MMRs according to the averaging level of MMRs in China. Data from baseline survey were gathered between June 2009 and October 2011, and the follow-up survey was conducted one year later following the baseline interview.

Based on inclusion criteria below [11, 12], potential target households in the affected group were identified through the registration system of the County Maternal and Child Office (CMCHO), which routinely collects information on all cases of childbirth and maternal death in selected provinces. Once eligibility of households was confirmed and informed content was signed, the caregivers who took care of children were invited for a face-to-face and standardized interview with assistance of trained interviewers. The households who had childbirth but not a maternal death were chosen to match the affected households according to the criteria below [11, 12].

Inclusion criteria for affected group:

- Having a maternal death within 3 months before the interview; a woman who died during late pregnancy (≥28 gestational weeks) or within 42 days after giving birth was defined as maternal death, excluding causes not attributable to the pregnancy.

Matching criteria for control group:

- Experiencing childbirth within 3 months before the interview, and;
- Dwelling in the same village and;
- Having comparable economic status estimated by the village leaders and;
- Having the same household type prior to maternal death (with or without older children; nuclear or extended family) and;
- Having the same ethnicity, if possible.

A total of 530 households who experienced a maternal death were recorded by the CMCHO in selected provinces during the study period. Of them, 40.9% (217/530) were excluded due to not meeting the inclusion criteria (primarily due to the maternal death occuring prior to 28 gestational weeks). Of the 313 households that met the inclusion criteria, 37.6% (118/313) refused to participate in the study because of their grief. This left 195 households to be interviewed in the affected group, accounting for 62.3% of the eligible participants. A total 384 households who experienced childbirth but not a maternal death were selected as a matching control group of the affected households. This resulted in a ratio of 2:1 control: affected study group; it is noted that 6 households in the affected group were matched by the control group in the ratio of 1:1 because they lived in the remote areas with few residents. During the follow-up, 183 households in the affected group and 346 households in the control group completed the survey. The present study only considered these 529 households who completed both baseline and follow-up surveys.

### Data collection

The baseline and follow-up surveys applied same measurement tools to collect the data. The caregivers of the children were chosen as respondents to answer questions regarding children. Questions regarding husbands had to be answered by the husbands.

The questionnaire included questions pertaining to the household socio-demographic characteristics (age, gender, ethnicity, household size, etc.), whether the child had diarrhea or cough in the past two weeks, as well as questions concerning the educational attainment for the older children to the present). Body length for children aged under 24 months and height for children aged 24 to 59 months (Wujin Weighing Apparatus Factory, Changzhou, China), and weight for all the children aged 0 to 59 months (TH Leaguer Sensory Technology company, Shenzhen, China) were measured. Each measurement was conducted twice and the mean value were obtained. Raw data were converted to Z-score and categorized to indicate the status of child nutrition: stunting was defined as Z-score of length/height-for-age below -2; underweight was defined as Z-score of weight-for-age below -2; wasting was defined as Z-score of weight-for-height below -2. Malnutrition was defined as presence of any of these adverse nutritional statuses (a Z-score below -2) [13].

In addition, information was collected to assess the mental health and the quality of life of husbands who experienced a maternal death. Breslau’s 7-item screening scale was adopted to evaluate the husbands’ posttraumatic stress disorder (PTSD) [14]. That scale uses an event checklist of trauma exposure which is scored by the summation of positive responses (scores for each question was ranging from 0 to 7). A cutoff score of 4 was used in the study to balance the scale’s sensitivity and specificity [15]. Also, the quality of life on husbands was evaluated by the widely used EuroQol questionnaire (EQ-5D) [16, 17]. The EQ-5D is a two-part questionnaire. The first part was composed of five domains. Each domain was further divided to three levels: no problems, some problems, and major problems. For each domain, husbands were asked to choose the level that matched their mental health at the time of survey. Responses to the five domains were generated as an original EQ-5D score. While there was no Chinese value set but a Japanese value set available, the original EQ-5D score was converted to the EQ-5D index by using Japanese value set [18]. The second part of the EQ-5D was a visual analogue scale (VAS) which designed to measure the husbands’ self-rating of their health state. Presented with a vertical line, the end points represented “worst health” at 0, and “best health” at 100. The number on the scale chose by the husbands was their VAS score (EQ-VAS), which was between 0 and 100.

### Data analyses

The demographic characteristics were compared between the affected and control groups, using Chi-square and the Wilcoxon tests.

For children, each of eight binary outcomes, including stunting, underweight, wasting, malnutrition, having diarrhea in the past two weeks, having cough in the past two weeks, not attending school and dropping out of school was compared between two groups using logistic regression models with adjusting for child’s age, father’s age, father’s education and household income. To measure the change from baseline to follow-up between the affected and control groups, we used multivariate regression and logistic regression models with interaction terms of survey time (follow-up vs. baseline) and group (affected vs. control group).

For binary outcomes, the difference-in-difference (DID) is estimated by the interaction terms and interpreted as the ratio of the odds ratio ($e^{\beta_{3}}$) of outcome of interest at follow-up vs. baseline for the affected group compared to the odds ratio of outcome of interest at follow-up vs. baseline for the control group. For continuous outcomes, the difference-in-difference (DID) is estimated similarly and interpreted as the difference of difference (*α*_3_) in means between affected and control groups.

1. The following model was employed for binary outcomes:
  $$logit(E[Outcome])=\beta_{0}+\beta_{1}Time+\beta_{2}Group+\beta_{3}Time\times Group+Other\;Covariates$$
2. The following model was employed for continuous outcomes:
  $$E[Outcome]=\alpha_{0}+\alpha_{1}Time+\alpha_{2}Group+\alpha_{3}Time\times Group+Other\;Covariates$$

In addition, to examine whether the attrition rate had an impact on the estimate of main outcomes, a sensitivity analysis using inverse probability weighting was conducted. The 95% confidence intervals (excluding 1) for the ratio of odds ratio represent statistically significant DID for binary outcomes. The 95% confidence intervals (excluding 0) for the difference of difference in means represent statistically significant effects for continuous outcomes. The threshold significant level was set at p < 0.05. Epidata3.1 software package was used to input data. The SPSS 20.0 for Windows statistical software package was used in present study.

## Results

### Effect on index children

**Fig 1:**
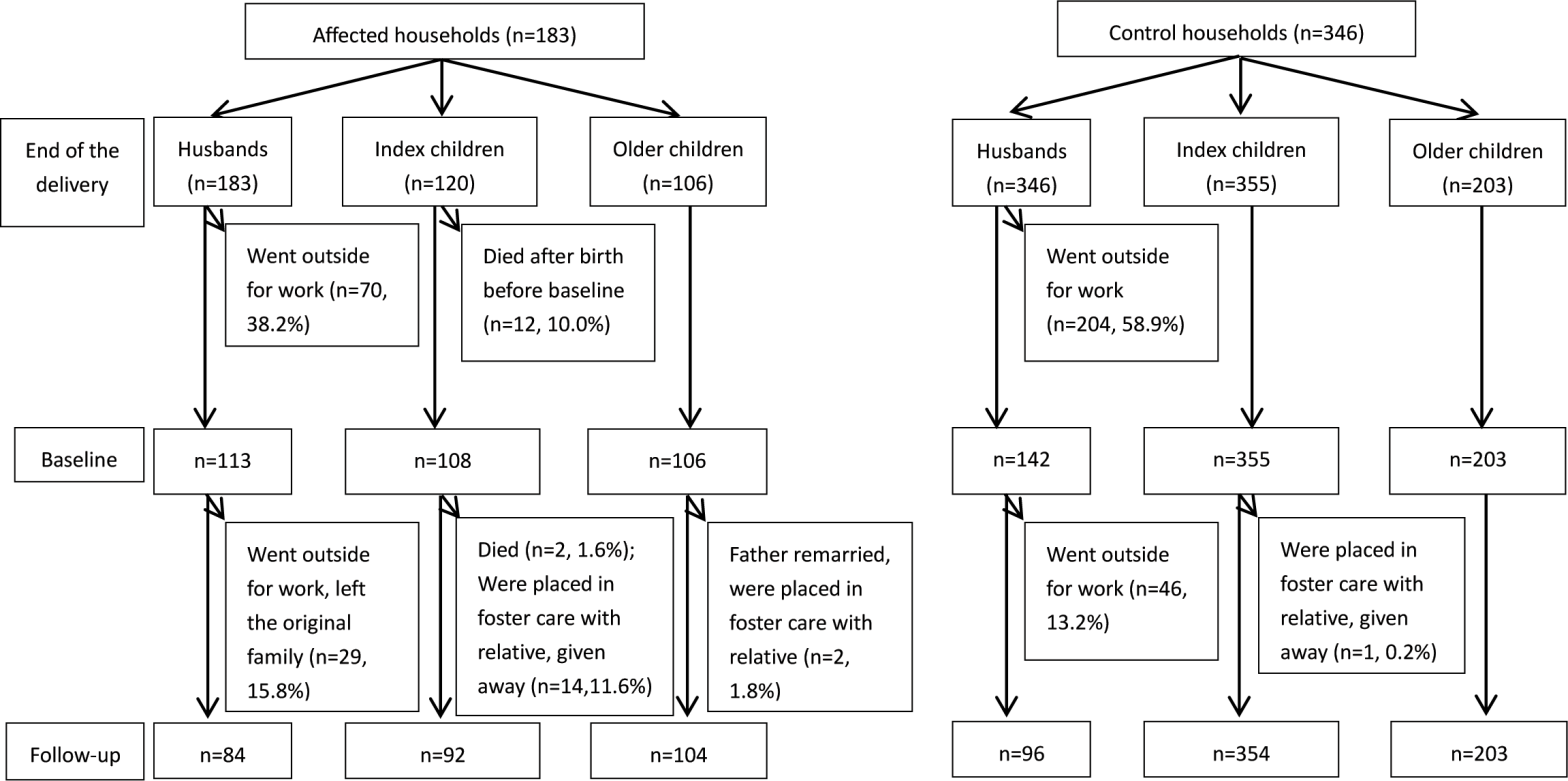
Flowchart of participants of affected households and control households. The figure represents selection of study participants in both groups from pre-baseline (end of the delivery) to baseline and follow-up. The primary reason of loss to follow-up for husbands is they went out for work during surveys. Index children in the affected group had a higher rate of death and abandonment than control group during pre-baseline and follow-up periods.

As shown in Fig 1, 120 index children from 183 households who experienced a maternal death and 355 index children from 346 households who had childbirth but not a maternal death were included in the analysis. A total of 14 of 120 index children (11.6%) died in the affected group, 12 of whom died before the baseline survey and 2 of whom died during the follow-up, and none died in the control group. Similarly, 14 of index children (11.6%) were abandoned in affected group in contrast to only one abandoned in the control group. Demographic characteristics including child’s gender, parents’ ethnicity, household size and family type and the total index children were comparable between two groups at baseline (Table 1). Median age of the index children in the affected group was one month older than those in the control group (3 months vs. 2 months).

**Table 1:** Characteristics of family members and households at baseline in 2009–2011.

Variables	Affected group			Control group		
	Responders	Non-responders	Total	Responders	Non-responders	Total
**Characteristics of index children**						
Age (Month) (median, IQR)	3 [2, 4]	-	3 [2, 4] ^b^	2 [2, 3]	-	2 [2, 3]
Gender (%, n/N)						
Male	45.4% (49/108)	-	45.4% (49/108)	47.6% (169/355)	-	47.6% (169/355)
Female	54.6% (59/108)	-	54.6% (59/108)	52.4% (186/355)	-	52.4% (186/355)
**Characteristics of older children**						
Age (Month) (median, IQR)	74 [51, 112]	-	74 [51, 112] ^b^	65 [42, 91]	-	65 [42, 91]
Gender (%, n/N)						
Male	51.9% (55/106)	-	51.9% (55/106)	47.8% (97/203)	-	47.8% (97/203)
Female	48.1% (51/106)	-	48.1% (51/106)	52.2% (106/203)	-	52.2% (106/203)
**Characteristics of husbands**						
Age (Years) (median, IQR)	33 [27, 39] ^a^	29 [26, 33]	30 [27, 36] ^b^	28 [24, 32]	28 [24, 32]	28 [24, 32]
Ethnicity (%, n/N)						
Han	76.2% (64/84)	83.8% (83/99)	80.3% (147/183)	63.5% (61/96) ^c^	86.4% (216/250)	80.1% (277/346)
Minorities^d^	23.8% (20/84)	16.2% (16/99)	19.7% (36/183)	36.5% (35/96)	13.6% (34/250)	19.9% (69/346)
Education (%, n/N)						
Illiterate	4.8% (4/84) ^a^	5.1% (5/99)	4.9% (9/183) ^b^	2.1% (2/96) ^c^	0.8% (2/250)	1.2% (4/346)
Primary	58.3% (49/84)	28.3% (28/99)	42.1% (77/183)	43.8% (42/96)	29.2% (73/250)	33.2% (115/346)
Secondary or higher	36.9% (31/84)	66.7% (66/99)	53.0% (97/183)	54.2% (52/96)	70.0% (175/250)	65.6% (227/346)
**Household characteristics**						
Number of children						
1	28.6% (24/84) ^a^	43.4% (43/99)	36.6% (67/183)	39.6% (38/96)	42.4% (106/250)	41.6% (144/346)
2 or more	71.4% (60/84)	56.6% (56/99)	63.4% (116/183)	60.4% (58/96)	57.6% (144/250)	58.4% (202/346)
Household size (%, n/N)						
1–5	90.5% (76/84) ^a^	72.7% (72/99)	80.9% (148/183)	78.1% (75/96)	77.2% (193/250)	77.5% (268/346)
6+	9.5% (8/84)	27.3% (27/99)	19.1% (35/183)	21.9% (21/96)	22.8% (57/250)	22.5% (78/346)
Nuclear families (%, n/N)						
Yes	48.8% (41/84) ^a^	18.2% (18/99)	32.2% (59/183)	33.3% (32/96)	30.4% (76/250)	31.2% (108/346)
No	51.2% (43/84)	81.8% (81/99)	67.8% (124/183)	66.7% (64/96)	69.6% (174/250)	68.8% (238/346)
Income (median, IQR)	3131 [2278, 4828]	3585 [2194, 5325]	3379 [2250, 5112] ^b^	4149 [2571, 6597] ^c^	4899 [3210, 7553]	4737 [2991, 7496]
Expenditure (median, IQR)	3952 [2504, 7346]	4773 [2643, 7211]	4132 [2523, 7217] ^b^	4852 [3125, 8188]	5212 [3174, 8584]	5070 [3164, 8554]

Affected group: households who experienced a maternal death; control group: households who had childbirth but not a maternal death

a. significantly different from non-responders in affected group (p<0.05)

b. significantly different from the control group (p<0.05)

c. significantly different from non-responders in control group (p<0.05)

d. In China, “minority” means non-Han ethnicity, including Hui, Yi, Hani, etc.

At the baseline, the prevalence of malnutrition among the index children in the affected group and control group was 40.0% and 14.5%, respectively (Table 2). The risk of malnutrition among index children in the affected group was higher than those in the control group (adjusted OR = 4.1, 95% CI: 2.3–7.2). Although the prevalence of malnutrition in the affected group at follow-up survey decreased, the risk of malnutrition in the affected group was still marginally higher than those in the control group (adjusted OR = 1.6, 95% CI: 0.9–2.8). The prevalence of stunting, underweight and malnutrition for the control group at follow-up survey was higher as compared to baseline. Adjusted DID estimators showed that significantly decreased possibilities for stunting (adjusted ratio of OR = 0.3, 95% CI: 0.1–0.8), underweight (adjusted ratio of OR = 0.2, 95% CI: 0.1–0.8) and malnutrition (adjusted ratio of OR = 0.4, 95% CI: 0.2–0.9) in the affected group compared with the control group. There was no significant variation in prevalence of diarrhea or cough between the two groups in both baseline and follow-up survey (Table 2). The prevalence of cough both in the affected and the control group increased at follow-up than at baseline.

**Table 2:** Health status of index children.

Outcomes	Baseline				Follow-up				DID		
	Affected n(%)	Control n(%)	OR (95%CI)		Affected n(%)	Control n(%)	OR (95%CI)		Adjusted^a^ OR _Follow-up vs. Baseline_		Adjusted^a^ Ratio of OR
			Unadjusted	Adjusted^a^			Unadjusted	Adjusted^a^	Affected	Control	
**Stunting**	28	29	5.1	5.0	27	64	1.9	1.7	1.0	3.0	0.3
	(32.9%)	(8.8%)	(2.8–9.3)	(2.6–9.4)	(32.5%)	(20.4%)	(1.1–3.2)	(0.9–3.0)	(0.5–1.9)	(1.8–4.9)	(0.1–0.8)
**Underweight**	16	13	5.6	5.1	7	19	1.4	1.0	0.4	1.6	0.2
	(18.8%)	(3.9%)	(2.6–12.5)	(2.3–11.7)	(8.4%)	(6.0%)	(0.5–3.4)	(0.4–2.6)	(0.1–0.9)	(0.8–3.4)	(0.1–0.8)
**Wasting**	5	16	1.2	1.4	2	4	1.9	0.9	0.4	0.3	1.6
	(5.9%)	(4.8%)	(0.4–3.2)	(0.4–3.8)	(2.4%)	(1.3%)	(0.3–10.1)	(0.1–5.7)	(0.1–1.9)	(0.1–0.7)	(0.2–11.3)
**Malnutrition**	34	48	3.9	4.1	28	69	1.8	1.6	0.7	1.8	0.4
	(40.0%)	(14.5%)	(2.3–6.7)	(2.3–7.2)	(33.7%)	(22.0%)	(1.1–3.0)	(0.9–2.8)	(0.4–1.4)	(1.2–2.7)	(0.2–0.9)
**Having diarrhea in the last two weeks**	20	64	1.0	1.0	19	60	1.0	1.0	0.9	0.9	1.0
	(18.5%)	(18.0%)	(0.6–1.8)	(0.6–1.8)	(17.6%)	(16.9%)	(0.6–1.8)	(0.5–1.7)	(0.5–1.9)	(0.6–1.4)	(0.5–2.3)
**Having cough in the last two weeks**	21	63	1.1	1.1	34	113	1.0	1.0	1.9	2.2	0.9
	(19.4%)	(17.7%)	(0.6–1.9)	(0.6–2.0)	(31.5%)	(31.9%)	(0.6–1.6)	(0.6–1.6)	(1.0–3.7)	(1.5–3.1)	(0.4–1.8)

Affected group: households who experienced a maternal death; control group: households who had childbirth but not a maternal death

a. Adjusted for child’s age, father’ age and education, and household income.

### Effect on older children

At follow-up, only two older children (1.8%) in affected group were abandoned and being cared for by relatives, as shown in Fig 1. The median age of the older children in the affected group (74 months) was larger than those in the control group (65 months).

As shown in Table 3, at follow-up survey, the risks of not attending school on time (adjusted OR = 6.8, 95%CI: 1.2–37.4) and dropping out of school (adjusted OR = 9.0, 95%CI: 1.4–56.2) in the affected group were higher than those in the control group despite no significant difference observed at baseline. The risk of cough was significantly higher in the affected group than those in the control group both in baseline and follow-up surveys. No significant differences were observed regarding risks of malnutrition and having diarrhea in the last two weeks between two groups in both baseline and follow-up surveys. The risk of stunting, underweight, malnutrition and prevalence of diarrhea and cough did not significantly change from baseline to follow-up period for each group.

**Table 3:** Heath status and educational attainment of older children.

Outcomes	Baseline				Follow-up				DID		
	Affected n(%)	Control n(%)	OR (95%CI)		Affected n(%)	Control n(%)	OR (95%CI)		Adjusted^a^ OR _Follow-up vs. Baseline_		Adjusted^a^ Ratio of OR
			Unadjusted	Adjusted^a^			Unadjusted	Adjusted^a^	Affected	Control	
**Stunting**	8	23	0.9	0.7	5	12	1.0	0.9	0.5	0.5	1.1
	(21.6%)	(24.2%)	(0.3–2.1)	(0.3–1.9)	(13.5%)	(14.0%)	(0.3–2.8)	(0.3–3.3)	(0.1–1.9)	(0.2–1.0)	(0.3–5.0)
**Underweight**	3	7	1.1	0.7	2	4	1.2	0.8	0.6	0.6	1.0
	(8.1%)	(7.4%)	(0.2–4.2)	(0.1–3.7)	(5.4%)	(4.7%)	(0.2–6.3)	(0.1–5.9)	(0.1–4.4)	(0.2–2.4)	(0.1–10.0)
**Wasting**	0	2	-	-	0	0	-	-	-	-	-
	(0.0%)	(2.1%)			(0.0%)	(0.0%)					
**Malnutrition**	8	24	0.8	0.7	6	12	1.2	1.2	0.7	0.4	1.5
	(21.6%)	(25.3%)	(0.3–2.0)	(0.3–1.9)	(16.2%)	(14.0%)	(0.4–3.4)	(0.3–4.0)	(0.2–2.3)	(0.2–1.0)	(0.4–6.3)
**Having diarrhea in the last two weeks**	8	13	1.1	1.2	6	11	1.0	0.9	0.7	0.8	0.9
	(7.5%)	(6.7%)	(0.4–2.8)	(0.5–3.2)	(5.7%)	(5.7%)	(0.3–2.7)	(0.3–2.7)	(0.2–2.2)	(0.4–1.9)	(0.2–3.5)
**Having cough in the last two weeks**	34	43	1.7	2.0	29	27	2.3	2.2	0.8	0.6	1.4
	(32.1%)	(22.2%)	(1.0–2.8)	(1.1–3.5)	(27.6%)	(14.0%)	(1.3–4.3)	(1.2–4.2)	(0.4–1.5)	(0.3–1.0)	(0.6–3.1)
**Not attending school on time**	3	3	1.7	2.8	6	2	5.4	6.8	2.4	0.6	3.4
	(4.3%)	(2.7%)	(0.3–8.5)	(0.5–16.3)	(8.8%)	(1.8%)	(1.1–27.4)	(1.2–37.4)	(0.6–12.9)	(0.1–4.2)	(0.3–35.0)
**Dropping out of school**	3	2	2.5	3.5	5	2	4.4	9.0	1.9	1.0	1.8
	(4.3%)	(1.8%)	(0.4–19.5)	(0.5–24.1)	(7.4%)	(1.8%)	(0.8–23.4)	(1.4–56.2)	(0.4–10.0)	(0.1–7.5)	(0.1–22.5)

Affected group: households who experienced a maternal death; control group: households who had childbirth but not a maternal death

a. Adjusted for child’s age, father’ age and education, and household income.

### Effect on husbands

The attrition rates for the husbands of the affected group at baseline and follow-up survey were 38.2% (70/183) and 15.8% (29/183), respectively. In the control group, the attrition rates at baseline and follow-up survey were 58.9% (204/346) and 13.2% (46/346), respectively. The main reasons for loss to follow-up were that the husbands were working outside or not at home at the time of the survey. The husbands in the affected group were more likely to be older, illiterate and have lower income than those in the control group.

The results of quality of life for husbands are shown in Table 4. At baseline, significant differences were seen in the EQ-5D index and EQ-VAS between the affected and control groups (EQ-5D index: 0.73 vs. 0.82; EQ-VAS: 50.1 vs. 83.1). At follow-up survey, the affected group reached a mean EQ-5D index of 0.78 and a mean EQ-VAS of 63.8; these differences in scores for the index group of 0.04 and 15.1 respectively are significantly lower than the control group. However, the control group remained unchanged while the affected group increased in terms of EQ-5D and EQ-VAS from baseline to follow-up. Adjusted DID estimators showed a significant increase for EQ-5D (0.04, 95%CI, 0.02–0.07) and EQ-VAS (15.6, 95%CI, 8.9–22.4) in the affected group compared with that in the control group. By comparing socioeconomic status at baseline, we found that there were some differences between husbands who stayed at home (responders) and those who went outside for work (non-responders) within each group, as shown in Table 1. To examine whether the high attrition rate had an impact on the estimate of main outcomes, a sensitivity analysis using inverse probability weighting was conducted. Results showed that after weighting, score of EQ-VAS was still significantly lower in affected group than that in the control group (Δbaseline -33.0 before weighting vs. -32.9 after weighting, Δfollow-up -17.8 before weighting vs. -17.6 after weighting), which indicated that the non-response may have minimal effects on the outcomes.

**Table 4:** Effect on the quality of life and mental health of husbands.

Outcomes	Baseline				Follow-up				DID		
	Affected (mean±SD)	Control (mean±SD)	Affected vs. Control Difference of Mean (95% CI)		Affected (mean±SD)	Control (mean±SD)	Affected vs. Control Difference of Mean (95% CI)		Follow-up vs. Baseline Adjusted^a^ Difference of Mean (95% CI)		Adjusted^a^ DID (95% CI)
			Unadjusted	Adjusted^a^			Unadjusted	Adjusted^a^	Affected	Control	
EQ-5D index	0.73±0.07	0.82±0.05	-0.09	-0.09	0.78±0.07	0.83±0.04	-0.05	-0.04	0.05	0.01	0.04
			(-0.11 –-0.08)	(-0.11 –-0.08)			(-0.07 –-0.03)	(-0.06 –-0.02)	(0.03–0.07)	(-0.01–0.02)	(0.02–0.07)
EQ-VAS	50.1±21.1	83.1±14.7	-33.0	-31.7	63.8±21.4	81.5±13.4	-17.8	-15.1	14.0	-1.2	15.6
			(-37.4 –-28.6)	(-36.4 –-26.9)			(-23.0 –-12.6)	(-20.7 –-9.4)	(8.0–20.0)	(-4.9–2.4)	(8.9–22.4)

Affected group: households who experienced a maternal death; control group: households who had childbirth but not a maternal death

a^.^ Adjusted for husbands’ age and education, and household income.

**Fig 2:**
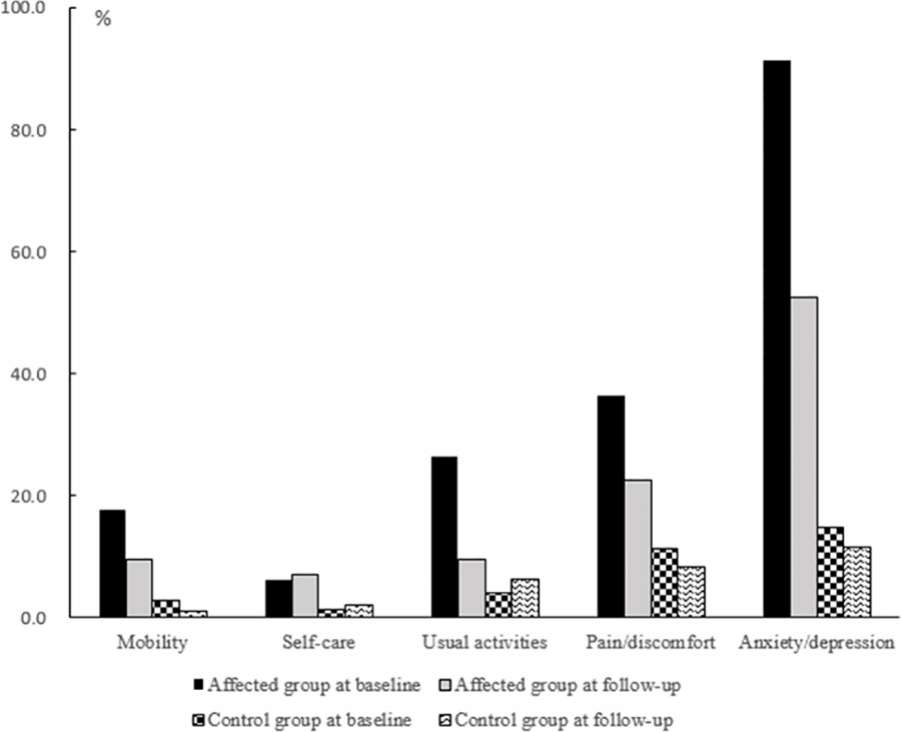
Assessment in each dimension of EQ-5D for the affected and control groups. The black and gray bars represent husbands in the affected group, and the other two bars represent those from the control group. In each domain of EQ-5Q, the higher score, the worse outcome it indicates.

Further analysis in each dimension of EQ-5D found that about 90% of the affected husbands at baseline had anxiety or depression (Fig 2), significantly higher than that in the control group (14.8%, p<0.001). Similarly, husbands in the affected reported a higher proportion of having pain or discomfort, and some problems doing usual activities at baseline than those in the control group (p<0.05). Approximately 20% had a problem with mobility and 6% had a problem with self-care at baseline in affected group. Although the reporting problems were reduced over time, more than half of the affected husbands still had anxiety or depression at follow-up survey, still significantly higher than that in the control group (p<0.001).

In addition, Posttraumatic Stress Disorder (PTSD) screening showed a significant decline from 4.71±2.00 at baseline to 3.85±2.00 at follow-up (p = 0.004) and the prevalence of PTSD decreased from 72.6% at baseline to 56.2% at follow-up (p<0.001) among husbands in the affected group, indicating the symptoms were modestly alleviated and the number of cases decreased during follow up period.

## Discussion

### Effect on index children

Our study found a relatively death rate (11.6%, 14/120) among index children within 15 months after maternal death, most of whom (85.7%, 12/14) died within 3 months after maternal death. This added evidence that maternal death was associated with elevated risk of dying at early stage among index children [16,19]. Of note, death rate among index children found in our study (11.6%) was lower than 48% reported in a study in rural Tanzania [6], possibly due to different health systems and cross-cultural differences, among other factors. Our study also found that index children who had experienced the loss of a mother had a significantly higher risk of malnutrition compared to those without experiencing the loss of a mother both at baseline and follow-up visits. Several possible reasons may explain elevated risk of malnutrition and dying among index children in the affected group.

First, early termination of breastfeeding makes babies vulnerable to malnutrition, which may contribute to the increased risk of infections or death [20–22]. Second, following maternal death, grieving husbands who used to earn money for family [23] may lack sufficient skills and responsibilities to take care of index children [24]. As shown in our study, 14 index children were abandoned by their fathers at the end of follow-up. Third, the economic burden due to the maternal death may act as a trigger to facilitate adverse effects on index children in the affected families [11,12,25].

Of note, our study also found that the prevalence of stunting, underweight and malnutrition among index children in the control group at follow-up survey was higher than at baseline. This may relate to inappropriate feeding practices adopted by caregivers in rural settings in China. Zhou and his colleagues found that the prevalence of stunting among children aged 6–36 months increased with age and the prevalence peaked at 25.4% among children aged 24–35 months in seven poor counties of China [26]. Early weaning, excessive water intake, too early or late intake of complementary foods and the inferior quality of complementary foods all contributed to increased risk of malnutrition [26].

### Effect on older children

Our results showed that the risk of not attending school on time and dropping out of school among older children in the affected households was higher than those in the control households at follow-up survey. This may derive from psychological vulnerability and bereavement reactions after experiencing the loss of a mother [27]. Children think about their deceased parent qualitatively differently than do adults who were related or knew the deceased. The psychological burden makes it harder for the children to concentrate on their schooling. Second, lack of prompt educational supervision and emotional support may be a barrier for education of orphaned older children [24, 28]. Third, older children among siblings may take caregiving responsibilities to look after their younger siblings or sick grandparents who lived with them, which may lead to early termination of education [27,29].

### Effect on husbands

Our study also found a significant poorer quality of life of husbands in the affected group compared to those in the control groups. According to National Health Services Survey (NHSS) of China in 2008, the mean VAS score of 58,163 men (aged 15–103) was 80.9 [30], which was similar to the level in the control group (83.1). However, it was much higher than the level in the affected group in this study, indicating that the quality of life of husbands was severely impacted by the death of their wife. Similar results found in a study in Sweden that men who lost a wife with cancer have increased risks of negative emotions including depression and anxiety compared with married men [31]. Starting a new relationship has been shown to be associated with a beneficial effect on the recovery for husbands who experience a maternal death [15], and thus increasing emotional support may improve mental health and quality of life of husbands suffering from maternal death.

Furthermore, our study found that over half of the affected husbands had a probability of having PTSD one year after maternal death. For husbands who experienced a maternal death in present study, PTSD may be result from not only the sudden loss of their wife, but also financial problems, additional caregiving responsibilities and coping with hardship in the family [30]. Utilizing collaborative care components and behavioral activation (BA) may be effective to attenuate PTSD among husbands as seen in veterans [32]. However, psychological counseling services are limited in rural China.

There are several limitations in our study. First, a high and different rate of loss to follow-up for husbands between the affected and control groups due to working outside home was seen. This might affect internal validity of estimates. More husbands in the affected group had to stay at home and take care of babies during the follow-up period, and more husbands in the control group went out for working in large cities, commonly practiced by families in rural China. Second, the absence of time trade-off (TTO) integrating conversion of EQ-5D for Chinese made it necessary to use a Japanese conversion. The difference between Chinese and Japanese is unknown, although researchers conducted a study demonstrating that the EQ-5D utility index is valid in Asian population [33]. Third, the duration of follow-up was relatively short and makes it impossible to observe the longer-term health effects on the children and husbands. Fourth, at the stage of the enrollment, about one third of participants, despite being eligible, refused to participate in the study because of their grief. This group might bear an even greater influence from the maternal death, which indicates we may report an underestimate of associations of maternal death with health outcomes of family members. Lastly, affected husbands were less educated, and lived in the household with less income than the control group at the baseline. Although our multivariate models had controlled for the differences in these baseline characteristics, there may some other unmeasured factors affecting the estimates.

In conclusion, our study demonstrates wide-ranging and spillover effects of maternal death on the physical and mental health of living children and husbands. Further reduction in the burden of maternal mortality is critical, and a public health attention should be paid to family members following a maternal death.

## Supporting Information

S1 Data (XLS)
